# The efficacy of collaborative psychological interventions in reducing anxiety levels in pregnant women: a systematic review and meta-analysis

**DOI:** 10.1186/s12884-025-07523-1

**Published:** 2025-04-11

**Authors:** Lely Firrahmawati, Widya Wasityastuti, Bernadette Josephine Istiti Kandarina, Eva Marti, Lukman Ade Chandra, Apri Sulistianingsih

**Affiliations:** 1 Doctoral Program in Medicine and Health Science, Faculty of Medicine, Public Health, and Nursing, Universitas Gadjah Mada, Yogyakarta, Indonesia; 2 Midwifery Study Programme, Faculty of Health Sciences, Universitas ’Aisyiyah Surakarta, Surakarta, Indonesia; 3 Department of Physiology, Faculty of Medicine, Public Health, and Nursing, Universitas Gadjah Mada, Yogyakarta, Indonesia; 4 Department of Biostatistics, Epidemiology, and Population Health, Faculty of Medicine, Public Health, and Nursing, Universitas Gadjah Mada, Yogyakarta, Indonesia; 5 Nursing Study Programme, Panti Rapih College of Health, Yogyakarta, Indonesia; 6 Department of Pharmacology and Therapy, Faculty of Medicine, Public Health, and Nursing, Universitas Gadjah Mada, Yogyakarta, Indonesia; 7 Midwifery Study Programme, Faculty of Health Sciences, Universitas Muhammadiyah Pringsewu, Lampung, Indonesia

**Keywords:** Anxiety, Pregnant woman, Psychological intervention, Collaboration

## Abstract

**Background:**

Anxiety during pregnancy can be harmful to both mother and baby, with anxiety rates remaining high despite psychological intervention efforts. This study aimed to evaluate the effectiveness of collaborative and single psychological interventions in reducing anxiety compared with standard antenatal care.

**Methods:**

A literature search was conducted in four databases (Scopus, Cochrane Library, PubMed, and ScienceDirect) for relevant studies published from 2016 to January 2024. The population in this review is pregnant women with anxiety who received psychological interventions either from a single health worker or involving collaboration. Psychological interventions were compared with standard antenatal care with maternal anxiety outcome scores to measure the efficacy of pre-post psychological intervention methods, with measurements taken only during pregnancy. The study designs included the use of the Randomized Controlled Trials method. This study restricted articles to languages ​​other than English and used a review design and pharmacological interventions. Two independent reviewers performed data extraction and quality assessment using RoB 2. Statistical analysis was conducted using R-Studio. Data analysis was performed using meta-count packages with a random effect model.

**Results:**

There were 14 eligible articles identified from the initial 3,346 records, with 1 article excluded from the meta-analysis. Psychological interventions were effective in reducing anxiety compared with standard care (Standardised Mean Difference (SMD) − 0.64, 95% CI − 0.98–−0.31). Analysis by type of intervention group showed differences between the two groups in mindfulness interventions (SMD − 0.55, 95% CI − 0.8–−0.31), motivational interviewing (SMD − 0.70, 95% CI − 1.08–−0.33), and supportive counseling (SMD − 0.73, 95% CI − 1.19–0.28). There were no differences between the Cognitive Behavioral Therapy (SMD − 0.80, 95% CI (− 1.80–0.19)) and Thinking Healthy Program intervention groups (SMD − 0.35, 95% CI − 0.81–0.11). Analysis of interventions conducted by a multidisciplinary team (collaborative) showed higher efficacy (SMD − 0.88, 95% CI − 1.60–−0.15) compared with a single professional (noncollaborative) (SMD − 0.47, 95% CI − 0.72–−0.22).

**Conclusions:**

Collaborative interventions show better efficacy than a single intervention, with psychological therapy being more effective in reducing anxiety rather than standard antenatal care. Future research should improve the cooperative approach and examine its long-term effects on maternal and newborn outcomes.

**Trial registration:**

This systematic review was registered in the International Prospective Register of Systematic Reviews (PROSPERO) under the registration code CRD42024497417.

## Introduction

Pregnancy anxiety is common and contributes to unfavorable delivery outcomes [1, 2]. A meta-analysis by Dennis et al. (2017) revealed a combined prevalence of clinically diagnosed anxiety disorders of 15.2% [3]. Similarly, a meta-analysis by Fawcett et al. (2019) showed a combined prevalence of prenatal anxiety of 20.7% [2]. Meanwhile, a more recent meta-analysis by Scott et al. (2022) showed that the total prevalence had increased to 29.2% [2]. However, many cases of anxiety are not correctly diagnosed, and the majority of pregnant women do not receive any treatment [4, 5]. High anxiety causes an increase in complex brain endorphins, which involves the release of corticotropin and cortisol hormones that interfere with the labor process as mediators in the placenta [6]. The effects of anxiety on pregnancy include premature birth, low birth weight, prolonged labor, postpartum depression, poor mother-infant bonding, and infant neurocognitive disorders [7–9].

Physiological and physical changes, family support, and social and emotional factors all cause anxiety in pregnancy [6, 10]. Good support and standardized pregnancy care are significant in reducing anxiety and fear before childbirth [11, 12]. Special attention to the mental health of pregnant women has become an important component of antenatal care, mainly because of the impact of anxiety on maternal and fetal health [5, 13, 14]. Various studies have shown that the integration of psychological services into antenatal care can achieve great success in overcoming anxiety [15]. For example, mindfulness-based interventions are effective in reducing anxiety and improving the overall well-being of pregnant women [16–20]. Other psychological interventions with psychoeducational models can reduce anxiety in pregnant women and prevent postpartum depression [21]. Research by Moyer et al. (2020) also supports the effectiveness of psychological interventions in reducing anxiety during the perinatal period, indicating that an integrated approach can provide better outcomes [11].

Antenatal care with an integrated psychological approach supports not only the mother’s physical health but also her mental health and social relationships, creating a supportive environment during pregnancy and the postpartum period [13]. Thus, the importance of integrating psychological services into antenatal care should be prioritized in clinical guidelines. The involvement of mental health professionals in collaboration with other health workers is effective in reducing anxiety and improving overall maternal well-being.

Recently, pregnancy care has developed through interprofessional collaboration with health workers such as psychologists, nurses, doctors, and midwives [22]. Interprofessional collaboration in this study is defined as a multidisciplinary approach in which various health professionals, such as psychologists, obstetricians, midwives, and nurses, work together in an integrated manner to design and implement interventions to reduce anxiety during pregnancy. The results of psychological intervention studies show that this intervention can have a significant impact on reducing anxiety in pregnant women [23]. Studies on different anxiety subjects and instruments note positive impacts, such as improving patient safety and quality of life, preventing medication errors, and improving the functional status of the patient [24]. Nevertheless, the many differences in pregnancy and delivery outcomes limit the benefits of collaborative care. A systematic review by Klatter et al. (2022) explained that collaborative interventions for the mental health of pregnant women used a lot of care but did not explain the means of communication between collaborative teams, offering limited evidence of a significant impact on pregnancy [25]. However, collaborative care continues to be developed to address the mental health of pregnant women. To our knowledge, there has been no systematic research or meta-analysis that compares interprofessional collaboration in addressing pregnancy anxiety. Previous studies have shown that perinatal anxiety has a significant impact on maternal and infant health, requiring different approaches to prevention and treatment. Several studies have evaluated the prevention of perinatal anxiety using interventions such as community-based mindfulness and social support. However, few studies have assessed the effectiveness of treatments in populations with a clinical diagnosis of anxiety. This study aimed to fill this gap by evaluating a collaboration-based intervention in the treatment of clinical anxiety during pregnancy. Therefore, it is essential to conduct a comprehensive meta-analysis to assess psychological interventions with collaboration and their impact on anxiety in pregnant women.

## Methods

### Objective

This study aims to conduct a systematic review with the latest meta-analysis of (1) This study evaluates the efficacy of collaborative psychological interventions, involving multidisciplinary teams, which are more effective than non-collaborative approaches for reducing anxiety levels in pregnant women. 2) The study examines whether pregnant women are compared to standard care (Standard antenatal care without the specified psychological interventions for anxiety). This systematic review was registered in the International Prospective Register of Systematic Reviews (PROSPERO; CRD42024497417). We used the Preferred Reporting Items for Systematic Reviews and Meta-Analyses (PRISMA) as a guideline for conducting the study [26–27].

### Data sources and search strategy

Two authors reviewed eligible studies and assessed the risk of bias using Cochrane guidelines [28], and extracted data. We searched four databases —Scopus, Cochrane Library, PubMed, and ScienceDirect— for relevant articles up to February 2024. The search strategy was not limited by language, publication time, or type of article. The literature search used Medical Subject Headings (MeSH). As for the keywords, we used the following search strategy to find more relevant publications: (1) mental health, (2) anxiety, (3) pregnant women, (4) collaboration, (5) interprofessional collaboration, (6) psychological intervention. We used the terms (“ “Pregnant Women” " [MeSH Terms] OR “pregnant women*” [Title/Abstract] OR (“Prenatal Care” [MeSH Terms] OR “prenatal care*” [Title/Abstract]) OR (“Anxiety” [MeSH Terms] OR “anxiety*” [Title/Abstract])) AND (“psychology, medical” [MeSH Terms] OR “psychology medical*” [Title/Abstract] OR (“Psychosocial Intervention” [MeSH Terms] OR “psychosocial intervention*” [Title/Abstract])).

### Eligibility criteria

In conducting this comprehensive meta-analysis, we systematically included English-language randomized clinical trials (RCTs) that specifically investigated the effects of psychological interventions on reducing anxiety in pregnant women compared with usual care. Nonrandomized trial design studies, observational studies, review articles, letters, and pharmacological interventions were excluded. In this study, we included RCTs that met the inclusion criteria that we had set: The population is pregnant women with anxiety who received psychological interventions either from a single health worker or involving collaboration, including midwives, doctors, psychologists, and nurses. The intervention was compared with standard antenatal care without the specified psychological interventions for anxiety. The “psychosocial intervention” keyword covers all psychological interventions given to pregnant women, including any behavioral interventions to reduce, change, or modify anxiety to improve mental health. We did not limit psychological interventions in any form. The outcome of this study was a decrease in maternal anxiety. The included research design used the Randomized Controlled Trials method. The eligibility criteria chosen for this study were based on the PICOS approach. This study measured efficacy using the pre-post intervention method, which was carried out only during pregnancy.

### Data extraction

Two independent reviewers screened titles and abstracts of articles for eligibility. Two authors conducted full-text screening based on the inclusion and exclusion criteria: differences of opinion were resolved through discussion and agreement with a third party. We used a self-completion form to collect information from the literature, including authors, year of publication, total number of study participants, sample size of the intervention and control groups, population characteristics (age, trimester of pregnancy), duration of study follow-up, and intervention characteristics (duration of intervention, type of intervention, number of intervention sessions, measurement tools for anxiety evaluation, and comparison characteristics [type of action taken, duration, and number of session]). The scope of this review is broad and includes all psychological interventions, so the purpose of the intervention, namely, preventing anxiety during pregnancy, is also recorded. This study also focuses on collaboration with health workers when conducting psychological interventions. This study extracted the mean anxiety scores and their standard deviations in the treatment and control groups. In the meta-analysis, we compared the differences in the Standardized Mean Difference (SMD) of the post-test anxiety scores of each group. In this meta-analysis, we excluded articles with post-test times that occurred postpartum.

### Outcome measures

The primary outcome included maternal anxiety after psychological intervention. We categorized it based on two subgroups: intervention type and collaboration or noncollaboration. Interventions with collaboration or interprofessional collaboration refer to cross-sectoral cooperation between two or more experts with different backgrounds and expertise to achieve common goals [24, 29, 30]. The types of psychological interventions included in this study are cognitive-behavioural, mindfulness, psychoeducation, MI psychotherapy, Thinking Healthy Program (THP), and counseling. Anxiety in pregnant women is defined as an affective condition involving worry and concern about fetal health, fetal loss, childbirth, and the care and treatment of the newborn [31]. We included studies with pregnant women who experienced anxiety as research subjects. Anxiety was determined using objective data based on the instruments used in each study. The included studies presented pre- and post-intervention anxiety score data (effect size calculations were available, namely mean and standard deviation) during pregnancy. The anxiety measurement instruments that were used are the Generalized Anxiety Disorder-7 (GAD-7), the State-Trait Anxiety Inventory (STAI), the HADS, and the Zung Self-Rating Anxiety Scale (SAS).

### Assessment of risk of bias

Each RCT study was assessed for bias risk using the Cochrane Risk of Bias Tool 2.0 criteria [32] outlined in the Cochrane Handbook for Systematic Reviews of Intervention [33]. The assessment was categorized as low risk, high risk, or moderate risk using the following domains: (1) randomization process; (2) deviations from the intended interventions; (3) missing outcome data; (4) measurement of the outcome; (5) selection of the reported result. Two reviewers, EM and LA, performed this assessment, and disagreements were resolved through discussion with a third party (WW and BI).

### Statistical analysis

We used R-Studio software (version 4.4.0) to conduct a meta-analysis with a random effects model that examined the standardized mean differences (SMD) in measuring anxiety levels after the intervention and postintervention follow-up [34]. Heterogeneity in these studies was evaluated using the I² statistic, which helps us understand how much results vary between studies. This random effects model was chosen because it allows us to consider variations between studies, especially those caused by differences in anxiety measurement scales.

SMD is usually interpreted using Cohen’s criteria: 0.2 indicates a small effect, 0.5 indicates a medium effect, and 0.8 or higher indicates a significant effect [35, 36]. This statistic is often interpreted as an indicator of heterogeneity ranging from low (25%) to medium/moderate (50%) and high (75%).

The degree of heterogeneity (i.e., τ2) was estimated using the restricted maximum likelihood estimator. The amount of heterogeneity (i.e., n τ2), estimated using the Q heterogeneity test and the I^2^ statistic, was also reported [37, 38]. The researcher set α = 0.05, so the value of ρ < 0.05 is considered significant.

This review analyzed a study comparing psychological interventions conducted collaboratively with psychological interventions without collaboration. It also analyzed studies comparing psychological interventions with standard care (without psychological intervention). The length of follow-up is considered in this review, so we analyzed subgroups of psychological interventions based on follow-up.

### Publication Bias analysis

Publication bias analysis was conducted using a funnel plot to evaluate the symmetry of SMD distribution against standard error. Interpretation to assess publication bias was done visually and statistically. Visual inspections checked any asymmetrical plot patterns, and Egger’s test evaluated the significant relationship between the effect size and standard deviation statistically. A p-value less than 0.05 confirmed the publication bias [39, 40].

## Results

**Fig. 1:**
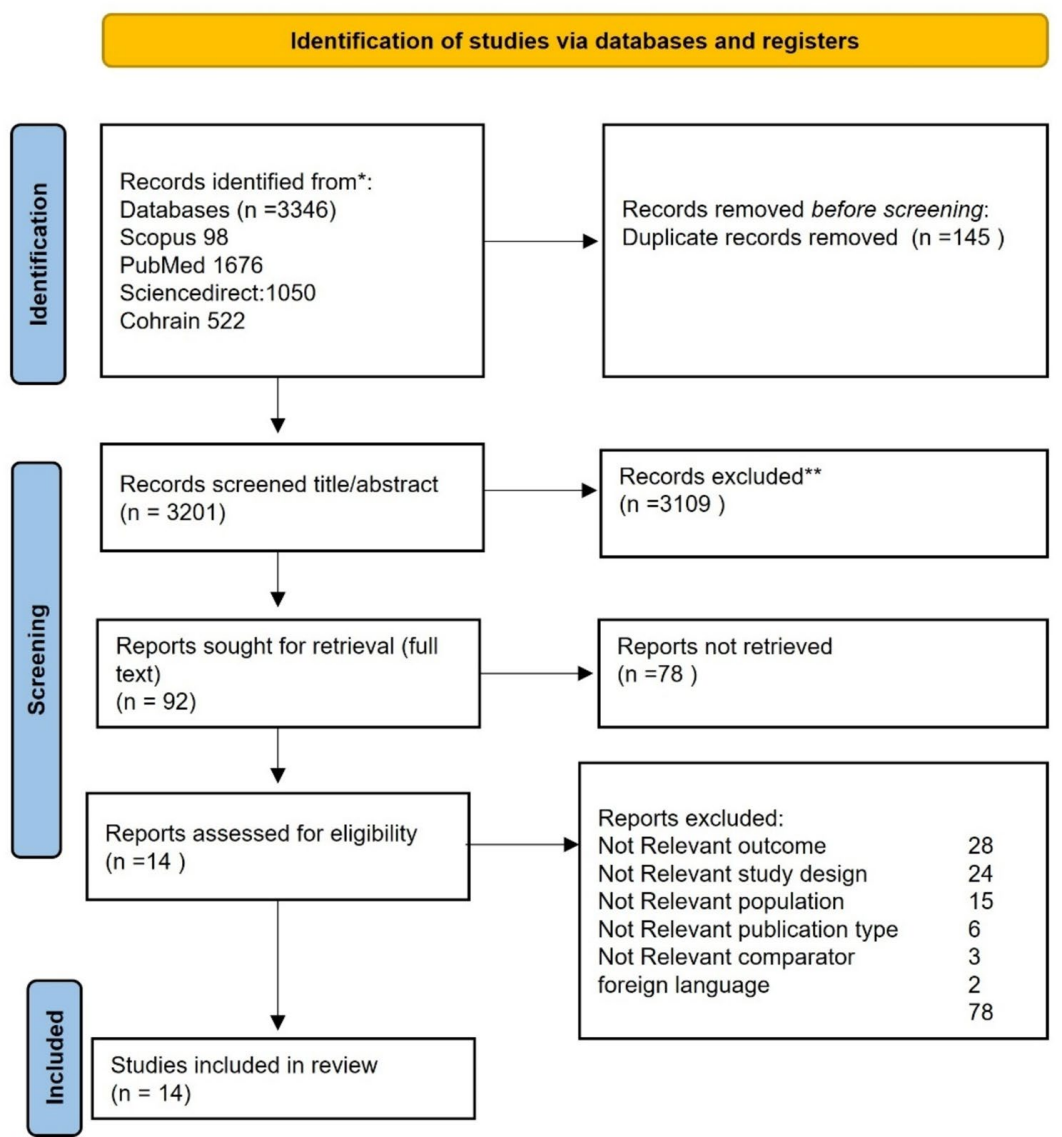
Flow of studies found by the systematic review

Our database search results yielded 3346 records from PubMed (1676), Scopus (1676), ScienceDirect (1050), and Cohrain (522). After the process of removing duplicate articles, 3201 records remained. Applying the inclusion and exclusion criteria reduced this number to 92. The researchers then excluded articles for the following reasons: (1) having different outcomes (*n* = 28); (2) different study designs (24); (3) different populations and no explanation of research criteria (*n* = 15); (4) inappropriate group comparisons (*n* = 2); and (5) untranslatable language (*n* = 3). Ultimately, 14 articles were included in the systematic review (Fig. 1).

### Study characteristics

#### Description of intervention methods

The intervention approaches reviewed consisted of six types: CBT, mindfulness, counseling, and Motivational Interviewing (MI) [41]. CBT interventions were used in four studies. Two articles used an online platform as the digital innovation, and the other two used individual meeting sessions [42–45]. All of these studies involved tasks for pregnant women to complete at home (Tables 1 and 2).

**Table 1:** **Overview and characteristics of included studies** (P**sychological interventions with collaboration)**

Author	Study location	Subject (Gestational Age) and Duration of Follow-Up	Number of sessions (hours)	Participants with anxiety risk	Primary intervention approach (Name) delivery format	Collaboration
Scherer, S 1 2016	Switzerland	18–32 weeks and with a duration of 6 weeks	Six core IB-CBSM modules	Yes	IB-CBSM	Collaboration: The therapist (trained psychologist or a psychologist-to-be under supervision) poses pregnancy-specific questions to the study team’s midwife.
Tam, Wing Hung 2023	China	< 34 - ≥37 weeks and with duration 6 weeks and 6 months postpartum	Ranged 1 to 4 sessions with a duration of 25–50 min	Yes	Educational counseling	Collaboration: The nurse and attending doctors were called to discuss about the obstetric management plan
Yang 1 2022	China	< 32 weeks and with a duration of 4 weeks	Participants in the *mindfulness* intervention groups received 1.5 h weekly *mindfulness* sessions for 4 weeks. intervention	No	*Mindfulness*	Collaboration: The content of each training program was validated by three experts, including a psychologist, an obstetrics expert, and a clinical nursing specialist. The psychologist contacted all participants during weeks 2 and 4 to answer questions about specific training problems, address difficulties, and encourage adherence. The midwifery and another nurse were responsible for WeChat group management.
Bayat1 2021	Iran	11–15 weeks and with a duration of 2 weeks	4 sessions of individual counseling	Yes	Cognitive- behavioral educations	Collaboration: certified midwife in cognitive-behavioral training under the supervision of a clinical psychologist.
Sun, Y 1 2021	China	12–20 weeks and with a duration of 8 weeks	25 min. The 8-week *mindfulness* training	No	*Mindfulness*	Collaboration: One obstetrician, One obstetric nurse and two research assistants with *mindfulness* experience participated.

**Table 2:** **Overview and characteristics of included studies** (P**sychological interventions without collaboration )**

Author	Study location	Subject (Gestational Age) and duration of follow-up	Number of sessions (hours)	Participants with anxiety risk	Primary intervention approach (Name) delivery format	Collaboration
Abdollahi 2020	Iran	26–33 weeks and with a duration of 5 weeks	120 min face-to-face group sessions for a period of 5 weeks. T	No	Motivational interviewing (MI)	Non-Collaboration: a midwife in the obstetrics clinic
Esfandiari, Maria 2020	Iran	6–32 weeks and with a duration of 6 weeks	for six sessions, once a week for two hours	No	Supportive Counseling	Non-Collaboration: Expert psychotherapist
Wang, Shui Lei 1 2023	Chinese	20–32 weeks and with a duration of 4 weeks	the first, second, and fourth weekends lasting 2.5 h and the third weekend lasting; After each on-site course, the intervention group practiced at home for 30–40 min per day, 6 Days a week, with the recorded audio by the WeChat 6.5 h. The total on-site intervention time was 14.	No	*Mindfulness*	Non-Collaboration: MBCP teachers with rich teaching experience
Burger 1 2020	The Netherlands	20–36 weeks and with a duration of 16 weeks	2 days of training on antenatal CBT (for depression, anxiety disorders, and PTSD) and supervision during	No	Cognitive–behavioural therapy (CBT)	Non-Collaboration: CBT therapists
Boran, P 2023	Istanbul, Turkey	12–30 weeks and with a duration of 6 weeks	intervention group sessions lasted one hour on average, except for the introduction session which lasted 90 min.	No	Thinking Healthy Programme (THP)	Non-Collaboration: antenatal nurses by trained THP supervisors Master Trainer (NA) online for approximately 1 h
Forsell 2017	Sweden	10–28 weeks and with a duration of 10 weeks	10 weeks later both online and with a telephone interview	No	ICBT	Non-Collaboration: CBT therapist
Sapkota 2022	Nepal	24–36 weeks and with a duration of 6 weeks	A single face-to-face counseling session in this study lasted 30 to 45 min.	No	Motivational Interview	Non Collaboration: Nurse
Zhang, Lin 1 2023	China	12–24 weeks and with a duration of 19 weeks	The 4-week *mindfulness*-based program involved weekly 30-minute sessions and 30–45 min per day of *mindfulness* practice.	No	*Mindfulness*	Non Collaboration
Zhang, Xuan 2 2023	China	12–20 weeks and with a duration of 25 weeks	On the first day of each week, participants viewed animated videos that provided thematic lessons for each module. Each video was 10- to 20-minute long a	No	*Mindfulness*	Non Collaboration

The operational definition of study characteristics that we included consisted of the author being the name of the principal investigator or study author responsible for the study. Study location is the location of the study, including country and institution, if relevant. Subject (Gestational Age) and duration of Follow-Up is the gestational age at which participants were recruited for the study and the duration of observation or follow-up after the intervention. Number of Sessions (Hours) is the number of intervention sessions conducted in the study, measured in time units such as minutes or hours. A participant with Anxiety Risk is whether the participant has a risk of anxiety (yes/no); based on the criteria or diagnosis used in the study, anxiety risk was determined based on the presence of prior obstetric complications, as reported in the included studies. These complications included factors such as a history of preterm birth, chromosomal abnormalities, or other significant obstetric events, which have been identified in the literature as contributors to heightened anxiety during pregnancy.

Primary Intervention Approach (Name) Delivery Format is the primary method of intervention used, such as Cognitive Behavioral Therapy (CBT), Mindfulness, or Motivational Interviewing, and the delivery format, such as face-to-face sessions, online or via an app. Collaboration is the involvement of collaborators in the study, including the types of professionals participating (e.g., psychologists, midwives, nurses) and their roles in supporting the implementation of the intervention.

Three studies had a population of pregnant women with anxiety risk factors. The population in the study [42] consisted of pregnant women who had positive results on chromosome screening for chromosomal disorders in the first trimester, indicating that they have a higher risk for anxiety disorders. The population recruited in [46] were women with disorders during pregnancy and childbirth, such as antenatal complications, instrumental delivery, elective or emergency cesarean section, and postnatal complications. This study identified articles that explained the risk factors for anxiety because respondents faced situations that could increase psychological vulnerability, including postpartum anxiety and depression. The study population [43] involved women with preterm labor (PTL), which is a significant medical condition during pregnancy.

**Table 3:** Characteristics of psychological interventions for perinatal anxiety in a nonanxious population

The intervention approach used in included studies	Studies selecting participants for anxiety risk	Studies assessing intervention modality components			
		Collaboration	Digital component	Mental health professional	Other health professionals or non-specialist provider
CBT	4	2	2	2	2
Mindfulness	5	2	5	1	3
Counseling	2	1	0	1	2
Motivational interviewing (MI)	2		1	1	1
Thinking Healthy Programme (THP)	1		0	0	1
Total	14	5	8	5	8

Counseling interventions were used in two studies, one using educational counseling and the other using supportive counseling [46, 47]. Mindfulness interventions were used in five studies, with most articles using digital innovations such as Smartphone-Based Mindfulness Training and WeChat to provide task guidance, notes, and daily mindfulness progress monitoring tools [18, 48–51].

The THP was used in one study conducted through online group sessions facilitated by midwives [52]. MI was used in two studies. The study by Abdollahi [41] used interview sessions with a psychotherapist and daily assignments recorded at home, and the study by Sapkota included an information booklet and a counsellor hotline that pregnant women could contact as needed [53]. Further details on the methods and results of each study can be found in Tables 1 and 2, and Table 3.

#### Description of interprofessional collaborations

There were five studies that involved collaboration between various health workers [18, 42, 43, 46, 48]. Two of these articles involved collaboration between midwives and clinical psychologists [42], one involved collaboration between nurses and obstetricians, another involved collaboration between psychologists, obstetricians, and clinical nurse specialists [39, 43, 44], and one involved collaboration between obstetricians, obstetric nurses, and two practitioners [18].

Nine studies did not involve collaboration or involved only one profession in implementing the intervention [41, 44–47, 49–53]. Seven studies involved psychotherapists in the intervention procedure, while two other articles involved nurses. These studies show a variety of intervention approaches used to improve maternal health through both cross-professional collaboration and interventions that focus on a particular profession. In this study, four articles used collaborative CBT interventions, and three studies did so without collaboration. Collaborative mindfulness interventions were recorded in five studies, while another five used mindfulness interventions without collaboration. All the studies that used MI were carried out without collaboration. Meanwhile, for the THP, only one study was carried out without collaboration.

### The risk of bias in the studies included

**Fig. 2:**
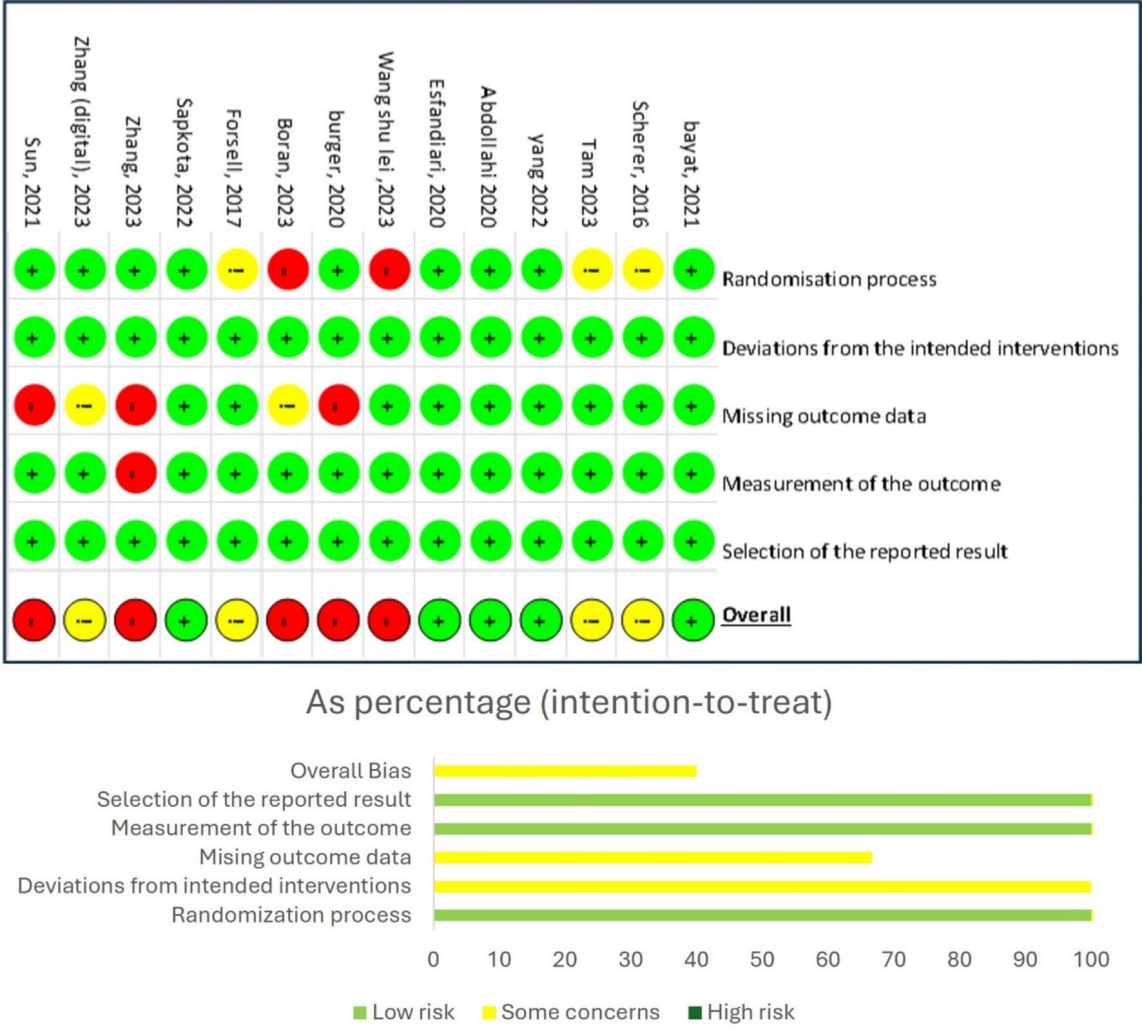
Risk of bias graph: reviewers’ domain assessments as study percentages

An assessment of the risk of bias showed five studies with low risk [41, 42, 47, 48, 53], four studies with moderate risk [43, 45, 46, 50], and five studies with high risk (Fig. 2). In selection bias, nine studies were at low risk, two studies [49, 52] were at high risk, and three [43, 45, 46] were at moderate risk. All studies had a low risk of performance bias. Most studies had complete outcome data. However, three studies [18, 44, 51] had a high risk of attrition bias, and two studies [50, 52] had a moderate risk of bias. Only one study [51] had a high risk of measurement bias. All studies showed a low risk of reporting bias.

The highest risk of bias was found in the randomization process, which had two high-risk articles and three medium-risk articles, and regarding incomplete data, with three high-risk and two medium-risk studies. Adequate Respondent assistance can increase commitment to completing the task and reduce the risk of bias due to missing data.

### Primary outcomes

#### Meta-Analysis

The total number of articles included in the meta-analysis was 13. Because there was one article that did not include post-test data during pregnancy, post-test data were available only after 32 weeks postpartum. These data did not match the PICOS of our meta-analysis, which only included anxiety measurements during pregnancy [46].

### Intervention effect

Anxiety in the intervention group compared with the control group.

**Fig. 3:**
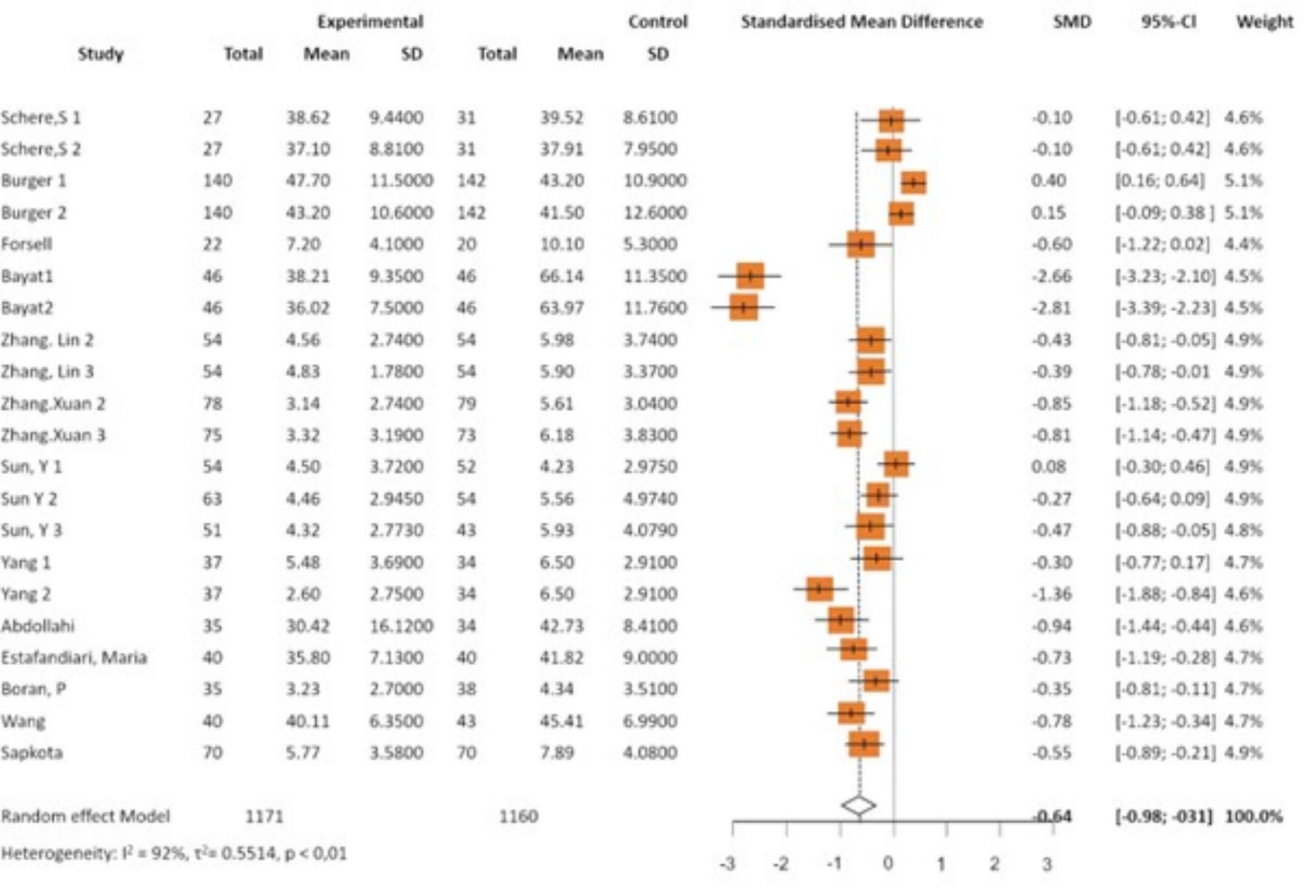
Forest plot showing anxiety outcomes of all interventions

The combined results of all interventions showed an overall negative effect size with SMD = − 0.64 (95% CI − 0.98–−0.31). This indicates that, on average, the psychological interventions significantly affected the intervention group compared to the control group. The high overall heterogeneity (I^2^ = 92%, *p* < 0,01) highlights the substantial variability in effect sizes across studies and interventions (see Fig. 3).

This variability highlights the importance of considering the context of individual studies when interpreting intervention effectiveness. Factors such as study location, implementation details such as intervention procedures, and participant characteristics (e.g., gestational age, age, and sociodemographic factors) should be considered to interpret the results better.

### Subgroup analysis

#### Psychological intervention type subgroup

We used meta-analysis to examine several moderators of the observed effects. Clustering analyses covered a range of intervention approaches. Psychological interventions with a collaborative approach are efforts that involve the collaboration of multiple health professionals, such as psychologists, nurses, doctors, and midwives, to provide psychological support to pregnant women. Examples of these collaborative approaches include THP, MI Psychotherapy, Cognitive Behavioral Therapy (CBT) delivered by a multidisciplinary team, mindfulness facilitated by multiple professionals, and supportive counseling that involves various experts. Meanwhile, noncollaborative interventions are approaches in which psychological support is provided by only one type of health professional without the involvement of a cross-professional team. Two examples are CBT, delivered only by a psychologist without collaboration with other professionals, and mindfulness, facilitated by a single practitioner without the involvement of other health professionals.

**Fig. 4:**
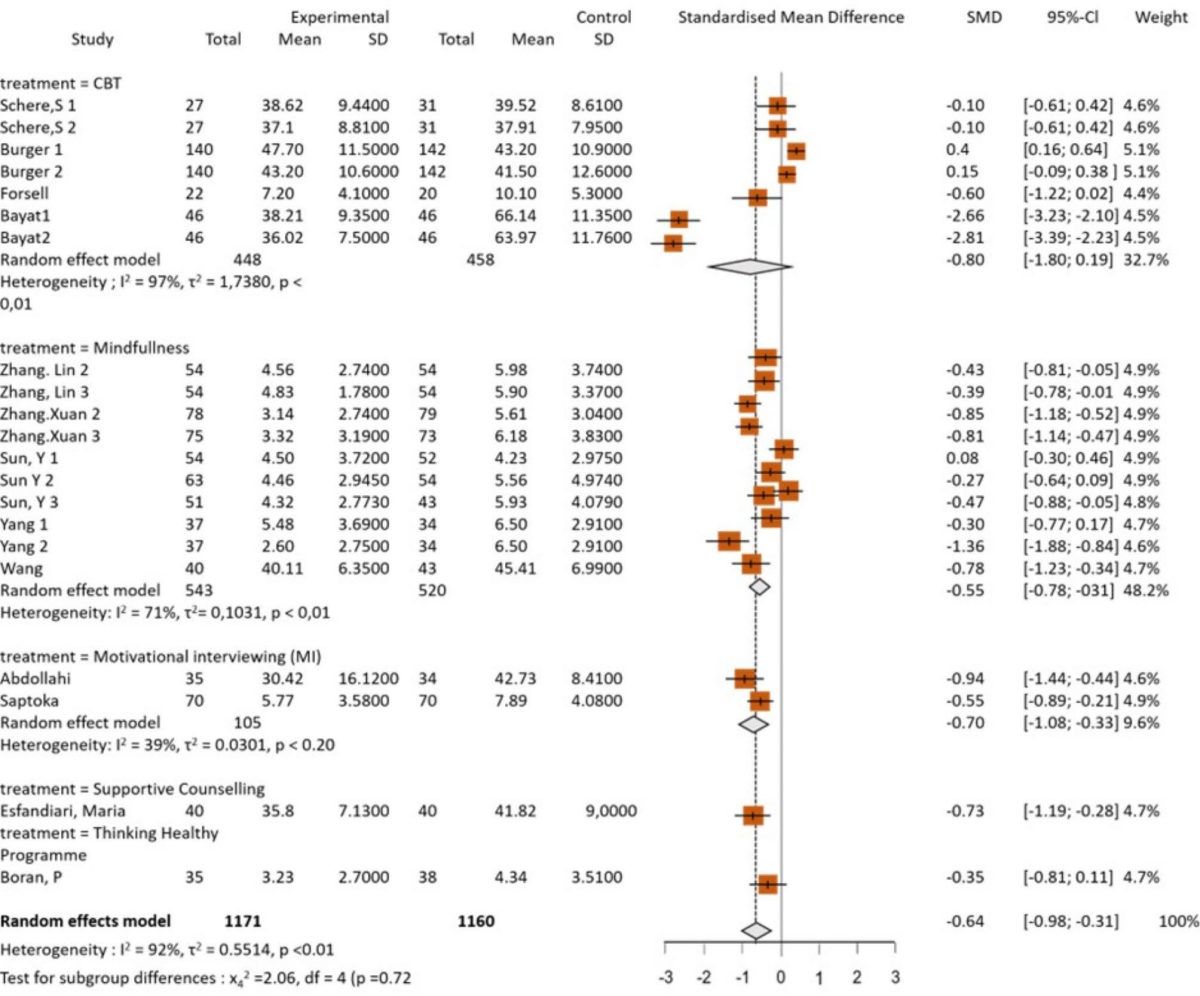
Forest plot showing anxiety outcomes from intervention subgroups in random effects model

Forest plots were used to show the comparative effectiveness of these psychological interventions, which included CBT, mindfulness, MI psychotherapy, supportive counseling, and the THP. SMD was used to measure effect size, which shows the difference in effectiveness between the control group and the experimental group in terms of the intervention (Fig. 4).

The overall random effects model for CBT showed a negative SMD (− 0.80; 95% CI − 1.80–0.19) and high heterogeneity (I^2^ = 97%). This indicates significant variability in effect sizes across studies, which suggests that CBT may be more effective in specific contexts or populations. Therefore, further investigation is needed to identify moderating factors.

The random effects model for mindfulness showed a negative SMD (− 0.55; 95% CI − 0.78–−0.31). These results suggest that mindfulness has moderate benefits compared with the control group. Moderate heterogeneity (I² = 71%, τ² = 0.1031, *p* < 0.01) indicates significant variation among the studies included. This suggests that the effects of mindfulness may differ depending on the context or the population studied.

A study conducted by Abdollahi [41] and Sapkota [53] evaluated the effectiveness of MI therapy. The results showed that the SMD was negative (− 0.70; 95% CI − 1.08–−0.33). This means that MI therapy has a large and significant effect on improving the condition of participants compared with the control group. Since the confidence interval does not include zero, this result is statistically significant, strengthening the evidence that MI therapy is effective. The global weight of this study was 9.6%, which indicates its substantial contribution to the entire meta-analysis.

According to Esfandiari, the effectiveness of supportive counseling was evaluated. The results showed that the SMD was negative (− 0.73; CI 95% −1.19–−0.28) [47]. This means that supportive counseling has a large and significant effect on improving the condition of participants compared with the control group. Because the confidence interval does not cross zero, this result is statistically significant, strengthening the evidence that supportive counseling is effective. The global analysis weight of this study was 4.7%, which indicates its essential contribution to the overall meta-analysis.

A study conducted by Boran evaluated the effectiveness of the Thinking Healthy Program (THP) [52]. The results showed that the SMD was negative (− 0.35, 95% CI − 0.81–0.11). This SMD value indicates a small effect size and is not statistically significant because the confidence interval crosses zero. This means that although there is an indication that the THP may have an effect, the result is not strong enough to state the effectiveness of this program with certainty. The weight of this study in the global analysis was 4.7%, which indicates its contribution to the entire meta-analysis.

### Collaboration subgroup

The studies included in the collaboration subgroup showed mixed results. The random effects model for the collaboration subgroup showed an overall SMD of − 0.88 (95% CI − 1.60–−0.15). Heterogeneity in this subgroup was very high, with an I² of 94%, a τ² of 1.1805, and *p* < 0.01, which indicates significant variation between studies.

**Fig. 5:**
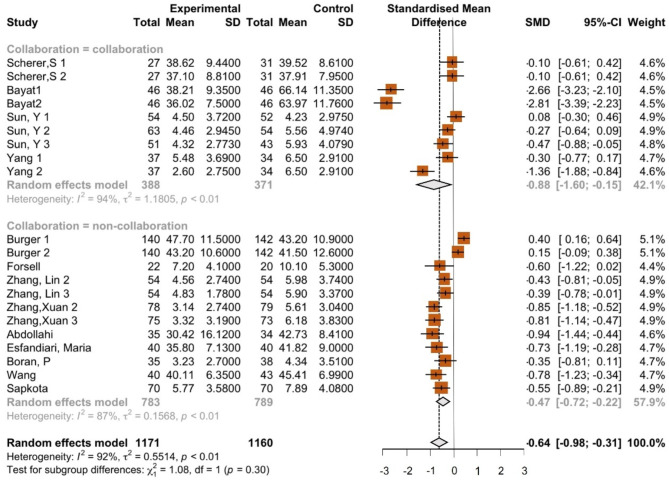
Forest plot showing anxiety outcomes by collaborative and noncollaborative treatments

The random effects model for the noncollaboration subgroup showed an overall SMD of − 0.47 (95% CI − 0.72–−0.22). Heterogeneity within this subgroup was also very high, with an I² of 87%, a τ² of 0.5514, and *p* < 0.01, which shows significant variation across studies. The SMD value in the collaboration group showed a more substantial effect size than the noncollaboration group and was statistically significant (Fig. 5).

### Length of follow-up

**Fig. 6:**
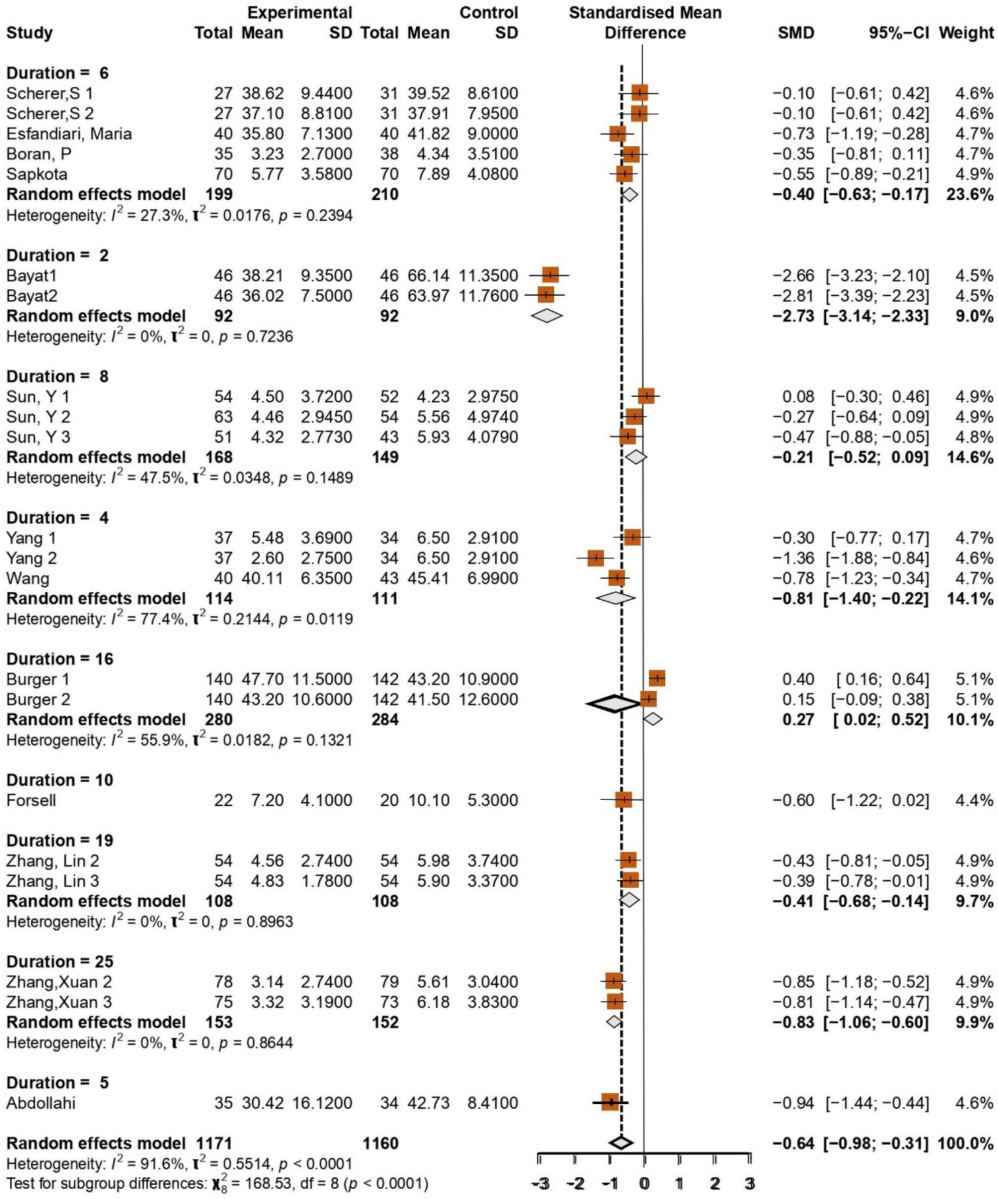
Forest Plot showing the duration of observation or follow-up after intervention

Duration = 8 (8 months follow-up) in Sun Y1, Sun Y2, Sun Y3 studies: SMD: 0.08 (CI: [-0.30, 0.46]), -0.17 (CI: [-0.64, 0.09]), and − 0.21 (CI: [-0.52, 0.09]) [18]. There are small and non-significant effects because the confidence interval includes zero. Moderate heterogeneity (I² = 47.5%), indicating moderate variation between studies. Duration = 4 (4 months follow-up) in Yang 1, Yang 2, Yang studies: SMD: -0.77 (CI: [-1.37, -0.17]), -0.80 (CI: [-1.24, -0.34]), and − 0.25 (CI: [-0.85, 0.34]) [48], Significant effects only in the first two studies; the latter effect was not significant. High heterogeneity (I² = 77.4%), indicating considerable variation between studies. Duration = 16 (16 months follow-up) Burger 1, Burger 2: SMD: 0.27 (CI: [-0.02, 0.52]) and 0.83 (CI: [0.39, 1.26]) [44], Small to medium effect, but only the second study was significant, Moderate heterogeneity (I² = 55.9%). Duration = 10 (10 Months Follow-Up) Forsell: SMD: -0.60 (CI: [-1.22, 0.02]), Medium effect but approaching significance [45]. Duration = 19 (19 months follow-up) Zhang Lin 2, Zhang Lin 3: SMD: -0.39 (CI: [-0.77, -0.01]) and − 0.30 (CI: [-0.56, -0.05]), Small but significant effect [51]. There is no heterogeneity between studies (I² = 0%). Duration = 25 (25 months follow-up) Zhang Xuan 2, Zhang Xuan 3: SMD: -0.85 (CI: [-1.18, -0.52]) and − 0.66 (CI: [-0.94, -0.37]) [50]. Significant and moderately large effect. Low heterogeneity (I² = 0%). Duration = 5 (5 months follow-up) Abdollahi: SMD: -0.64 (CI: [-0.84, -0.44]) [41]. Significant effect with moderate strength. Overall, the pooled SMD value was − 0.64 [95% CI: -0.96, -0.31], indicating a significant effect with a negative direction. The total heterogeneity was I² = 91.6%, indicating a high variation between studies. The subgroup difference test showed a significant difference between groups based on duration (*p* < 0.0001). The SMD values ​​ranged from − 0.85 to -0.66, with the 25-week duration group showing a more significant effect size than the other groups and was statistically significant. The longest duration (25 months) follow-up durations tended to have more significant effects than moderate durations (Fig. 6).

#### Publication Bias analysis

**Fig. 7:**
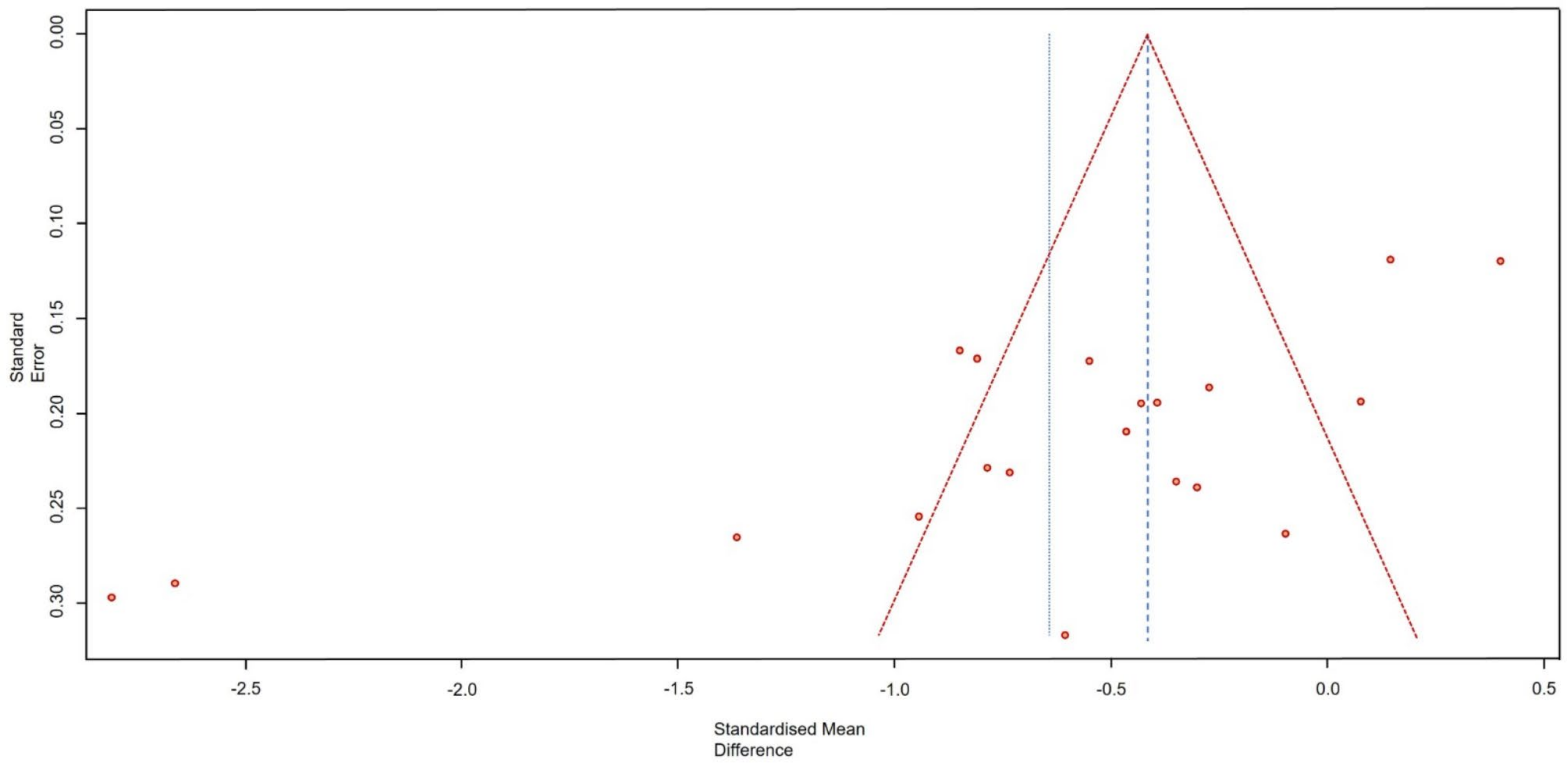
Funnel plot

The results of the funnel plot demonstrated a relatively asymmetrical distribution pattern, indicating the presence of publication bias. Visually, Fig. 7 reveals an imbalanced funnel plot, with a differing number of studies on each side (12 versus 8 studies). Moreover, several studies, both small and large in size, were outside the funnel triangle, reflecting varying and imprecise effect sizes. Notably, the number of studies outside the triangle was greater on one side. This visual indication of publication bias was further confirmed through statistical analysis using Egger’s test, which yielded a p-value of 0.0009.

## Discussion

This comprehensive review examines the effectiveness of collaborative interprofessional psychological interventions aimed at reducing anxiety in pregnant women. Our systematic review and meta-analysis have identified important trends and specific intervention outcomes that significantly affect clinical practice and future research.

The results of this systematic review and meta-analysis indicate that psychological interventions are effective in reducing anxiety in pregnant women, with mindfulness-based interventions (MBIs), supportive counseling, and MI showing the most significant impact. These findings corroborate previous research, which has also highlighted the efficacy of nonpharmacological interventions for managing perinatal anxiety, offering women an alternative to pharmacological treatments that may not be appropriate during pregnancy because of potential adverse effects on the mother and fetus [1, 18].

In this review, mindfulness-based approaches emerged as consistently effective, which is in line with previous research that highlights their benefits in reducing anxiety, depression, and stress across a range of populations [48]. MIs promote awareness and regulation of emotions and reduce cortisol levels and stress, making them particularly suitable for pregnant women who experience fluctuating anxiety due to hormonal and physiological changes during pregnancy [54].

CBT, a widely recognized intervention for generalized anxiety disorder, has shown mixed results. Although it is effective in treating anxiety in specific settings, this review revealed that the impact of CBT on pregnant women was inconsistent, depending on factors such as delivery mode (face-to-face vs. online) and treatment duration [43]. The emotional needs of pregnant women, especially those in the third trimester, suggest that CBT may require adaptation to be more effective for this population [45].

Supportive counseling and MI have also been identified as effective interventions, particularly in collaborative care settings. A collaborative approach with an interdisciplinary team that includes midwives, obstetricians, and psychologists provides comprehensive support that meets participants’ emotional and physical needs [41, 47]. The results of this study highlight the value of teamwork in treating pregnant women with psychological interventions, as collaborative interventions have been shown to enhance benefits and reduce anxiety more effectively than stand-alone interventions [53].

Anxiety during pregnancy has wide-ranging consequences for maternal and fetal health. Studies consistently show that untreated anxiety in pregnant women leads to adverse perinatal outcomes such as preterm birth, low birth weight, and developmental delay in children [7]. In this review, high levels of anxiety during pregnancy were associated with an increased stress response, which triggers neuroendocrine changes that are detrimental to the pregnancy process. These neuroendocrine changes include the release of corticotropin-releasing hormone and cortisol, both of which affect fetal development [6].

The significant reductions in anxiety demonstrated by MBI, supportive counseling, and MI across studies suggest that these interventions are effective in reducing the impact of anxiety on pregnancy outcomes. Managing anxiety through psychological interventions contributes to improved maternal-fetal bonding, better mental health after delivery, and a lower risk of postpartum depression [3, 8].

Efforts to address anxiety during pregnancy have evolved significantly over the years. The potential risks of traditional pharmacological treatments such as anxiolytics and antidepressants have led to the development of nonpharmacological interventions, whose use continues to increase. MBIs have been particularly successful because of their adaptability and accessibility [48]. Other interventions, such as CBT and MI, are also significantly more effective when they are implemented through interdisciplinary care models [54].

Supportive counseling has emerged as a key intervention, providing a platform for pregnant women to express their fears and concerns in a safe environment. This counseling helps manage specific stressors related to pregnancy and, when integrated into prenatal care, can significantly reduce anxiety levels [47]. Collaborative care models that combine psychological support with physical care have been shown to be particularly beneficial, allowing for a more holistic approach to maternal health [43].

One unique aspect of this study is its focus on the collaborative nature of the interventions delivered. Integrating diverse professional expertise—from psychologists and psychiatrists to obstetricians and midwives—creates a comprehensive support system that addresses multiple aspects of maternal anxiety. For example, an intervention that involves midwives and obstetricians and ensures that psychological care is seamlessly integrated into routine prenatal care. It also reduces the stigma associated with seeking mental health care [55–57]. Furthermore, the novelty of this study lies in its analytical approach, which focuses on the influence that different collaborative practices have on the effectiveness of the intervention. Using robust statistical analysis, we have described the specific contributions of each professional group involved in the intervention, providing a nuanced insight into the kinds of collaborations that are the most beneficial in particular contexts.

This study builds on the foundation laid by previous systematic reviews, which have focused primarily on the effectiveness of psychological interventions for managing anxiety and stress during pregnancy. These reviews have highlighted the benefits of MBIs, CBT, and supportive interventions across populations, but few have directly compared collaborative and noncollaborative models of these interventions [1, 18]. Furthermore, while previous studies have acknowledged the benefits of psychological interventions, their conclusions about long-term effects and cross-cultural applicability have been limited. This systematic review is novel in its comprehensive comparison of psychological interventions, particularly in its analysis of the impact of collaborative care models. The findings suggest that collaborative interventions have a more profound effect on reducing anxiety, a conclusion that has not been thoroughly explored in previous reviews [53].

The results of this study are consistent with previous research, which has consistently shown that nonpharmacological interventions are effective in reducing anxiety during pregnancy. MBIs have been widely reported to reduce anxiety and depression in a variety of populations including, pregnant women [48]. This study corroborates these findings, demonstrating that MBIs, particularly when integrated into collaborative care models, result in significant reductions in anxiety. CBT, although recommended for anxiety management in the general population, has shown mixed results in pregnant women. This review confirmed the variability in the effectiveness of CBT in this demographic, which suggests that CBT may require adaptation to address the unique emotional challenges of pregnancy [45]. Supportive counseling and MI were also confirmed as effective interventions when delivered through a collaborative care model that involves multiple health professionals [41].

One of the main strengths of this review is its comprehensive coverage, which addresses a range of psychological interventions and compares their effectiveness in collaborative and noncollaborative care models. The inclusion of RCT study designs provides a solid base of evidence for assessing the efficacy of psychological interventions in reducing anxiety during pregnancy. The focus on interdisciplinary collaboration also adds to the novelty of this review. By evaluating the impact of collaborative care on anxiety outcomes, this study provides new insights into healthcare team collaboration which delivers more effective psychological interventions. Including studies from various geographic areas and settings further strengthens the generalizability of the findings. Along with its strengths, this review has several limitations. The heterogeneity of the studies included presents challenges as variations in study design, population characteristics, and outcome measures complicate the direct comparison of results. The use of self-report measures to assess anxiety in most studies also introduces potential bias as participants may have under- or overreported their anxiety levels [6]. Another limitation is the lack of long-term follow-up data in many of the studies included. Although the interventions are effective in reducing anxiety during pregnancy, it is unclear whether these benefits persist after delivery or translate into better outcomes for mothers and infants [8]. Further research is needed to evaluate the long-term impact of these interventions on maternal mental health and child development.

The high heterogeneity observed in the results of our meta-analysis also underscores the complex nature of psychological interventions in pregnancy. This heterogeneity reveals the need for rigorous, context-specific research to refine these interventions further, ensuring they are tailored to diverse healthcare systems and cultural backgrounds.

The findings of this review have several important clinical implications. First, they highlight the need for healthcare providers to incorporate psychological interventions into routine prenatal care, specifically, MBIs, supportive counseling, and MI. These interventions have been shown to reduce anxiety effectively and can be offered as part of standard care for pregnant women who experience anxiety [1]. The importance of interdisciplinary collaboration is also emphasized. Healthcare providers, including midwives, psychologists, obstetricians, and social workers, should work together to provide holistic care that meets the psychological and physical needs of pregnant women. Collaborative care models have been shown to enhance the effectiveness of psychological interventions and should be encouraged in clinical practice [43]. Our study results support the effectiveness of a collaborative-based intervention for the treatment of clinical anxiety during pregnancy. Although our focus is on treatment, we acknowledge that prevention of perinatal anxiety is an important area that requires further research. Future studies could evaluate similar interventions in low-risk populations to assess their preventive potential.

The funnel plot visually and statistically indicated asymmetry in the results of the studies used in the analysis, with some studies being outside the confidence interval. This finding suggests the presence of publication bias in the study process, where certain results may have been omitted, thereby reducing the overall validity of the findings. However, this study acknowledges that the primary contributing factor is likely the high degree of heterogeneity among the included studies, derived from the considerable variation in intervention methods, measurements, and tools used.

Future research should focus on addressing the limitations of the current base of evidence. More high-quality RCTs are needed to confirm the effectiveness of psychological interventions, especially in diverse populations and settings. Studies should also include long-term follow-up to assess the ongoing impact of these interventions on maternal mental health and infant outcomes [8]. In addition, studies should explore the mechanisms by which these interventions reduce maternal anxiety. Understanding the factors that contribute to the success of these interventions, such as the role of interdisciplinary collaboration and the specific components, will help optimize their effectiveness and improve outcomes for pregnant women [54].

## Conclusions

This study demonstrates the effectiveness of psychological interventions in reducing anxiety among pregnant individuals and shows that collaborative models achieve better results than noncollaborative approaches These findings emphasize the need for interdisciplinary collaboration in prenatal mental health care and suggest that mindfulness, MI, and supportive counseling should be prioritized alongside CBT in clinical guidelines. The high variability in outcomes across interventions indicates the importance of tailoring approaches to individual needs. Future research should continue to explore the long-term benefits of these interventions and investigate the mechanisms by which they improve maternal mental health outcomes.

## Data Availability

The datasets used and/or analyzed during the current study are available from the corresponding author on reasonable request.

## Abbreviations

CBT: Cognitive Behavioral Therapy
GAD-7: Generalized Anxiety Disorder-7
RCT: Randomized clinical trials
HADS: Hospital Anxiety and Depression Scale
MI: Motivational Interviewing
PICOS: Population, Intervention, Comparative, Outcome, Study
SAS: Zung Self-Rating Anxiety Scale
STAI: State-Trait Anxiety Inventory
SMD: Standardised Mean Difference
THP: Thinking Healthy Program
